# Use of principal components to aggregate rare variants in case-control and family-based association studies in the presence of multiple covariates

**DOI:** 10.1186/1753-6561-5-S9-S29

**Published:** 2011-11-29

**Authors:** Rémi Kazma, Thomas J Hoffmann, John S Witte

**Affiliations:** 1 Department of Epidemiology and Biostatistics and Institute for Human Genetics, University of California San Francisco, 1450 Third Street, Box 3110, San Francisco, CA 94148-3110, USA

## Abstract

Rare variants may help to explain some of the missing heritability of complex diseases. Technological advances in next-generation sequencing give us the opportunity to test this hypothesis. We propose two new methods (one for case-control studies and one for family-based studies) that combine aggregated rare variants and common variants located within a region through principal components analysis and allow for covariate adjustment. We analyzed 200 replicates consisting of 209 case subjects and 488 control subjects and compared the results to weight-based and step-up aggregation methods. The principal components and collapsing method showed an association between the gene *FLT1* and the quantitative trait Q1 (*P*<10^−30^) in a fraction of the computation time of the other methods. The proposed family-based test has inconclusive results. The two methods provide a fast way to analyze simultaneously rare and common variants at the gene level while adjusting for covariates. However, further evaluation of the statistical efficiency of this approach is warranted.

## Background

With recent technological developments in human genome sequencing, enormous numbers of rare single nucleotide variants can now be detected. This ability to measure rare variants allows researchers to investigate the multiple rare variant/common disease model, which may help to elucidate part of the missing heritability in studies of more common variants. However, the low frequency of these rare variants raises issues about how best to analyze them [1]. In particular, the naive approach, which consists of testing each variant independently, has little power unless the sample sizes are large [2]. Instead, one can try to increase the statistical power by aggregating rare variants into meaningful groups (e.g., defined by genes, pathways, or functionality). Several approaches to combining rare variants have been proposed [2-9], but few address the issue of combining rare variants with common variants. The combined multivariate and collapsing (CMC) method consists of combining the test statistic of the collapsed rare variants with the univariate tests statistics of the common variants [3,6]. However, in practice one will often want to adjust for other covariates. When the number of common variants within a region is large, the logistic regression model may be unstable and may not fit well.

To overcome this problem, in this paper we propose two approaches, one for unrelated case and control subjects and one for families. The approaches aggregate rare and common variants into a single variable using a principal components analysis (PCA). These approaches are applied to case-control and nuclear family data sets from the simulations of Genetic Analysis Workshop 17 (GAW17) [10]. Using the case-control data sets, we also compare our method to a weight-based collapsing method adapted for quantitative phenotypes [9] and to the step-up approach [5].

## Methods

### Principal components and collapsing method

To test jointly the effect of rare and common variants with a case-control sample, we adapted the CMC method (described by Dering et al. [3] and Li and Leal [6]) by performing, within each gene, a PCA using one term for the aggregated score for rare variants and another term for each of the common variants. We call this the principal components and collapsing (PCC) method. The first principal component is then used in a linear regression model with other covariates. Combining the collapsed rare variants term and the common variants term into a single term often gives a more stable fit than the CMC approach. Collapsing the rare variants before the PCA allows rare variants to contribute some weight to the PCA and uses fewer degrees of freedom than the standard multimarker test collapsing such terms.

A similar method can also be used with a family-based sampling. Let *i* index the families, *j* the offspring, *c* the common markers, and *r* the rare markers, and let . Let *X_ijc_* and *X_ijr_* be the additive coding for common and rare genetic variants, respectively. For each gene, a PCA of all *X_ijc_* and of the sum over *r* of all *X_ijr_* is undertaken. Then, the numerator of our test statistic is given by:(1)

where *T_ij_* is the mean centered trait or residual from a regression analysis to adjust for covariates, *C_ij_* denotes the first principal component, and *P_ijk_* indicates the corresponding parental genotypes. This numerator can be derived as a score test from a conditional likelihood (as in [11] or [12]) and would be the standard family-based association test (FBAT) [13] if *C_ij_* was a single variant rather than the aggregated genetic score. To compute , we use Mendelian transmissions from the parental mating type, not population allele frequencies. Thus the test is robust to population substructure. An algorithm for computing  is given by Rabinowitz and Laird [14], along with an extension to more complicated family structures by conditioning on the sufficient statistic for parental genotype instead of the parental genotype (e.g., when parents are missing). The test can be thought of as a generalized covariance between the trait adjusted on covariates and the collapsed genetic score. The empirical variance of *S* is given by:(2)

Then the test statistic *S*/[var(*S*)]^1/2^ has a normal distribution.

### Weight-based collapsing

Another way to collapse variants is to weight them by allele frequency [3], a more appropriate model if less common variants are increasingly deleterious. To adjust for covariates, the phenotype is regressed on the nongenetic covariates, and then the residuals are used with the weight-based collapsing (WBC) method [9]. Briefly, the method weights by the inverse square root of the variance of the minor allele frequency (MAF) and then corrects by permutation.

### Step-up method

We also applied a separate data-driven collapsing method (the step-up [SUP] method) proposed by Hoffmann et al. [5]. Briefly, the approach uses a stepwise routine to determine the optimal grouping of rare and common variants. The initial step entails selecting among all variants of a group (in our case, a gene), the variant  with the strongest univariate test statistic (i.e., lowest *P*-value). Then all combinations of all other variants with  are tested jointly, and the combination  with the lowest *P*-value is selected as the best combination if its *P*-value is lower than the *P*-value of the test of  alone. The algorithm continues until all variants are included in the model or until all combinations of a rank *r* have a *P*-value higher than that of the previous rank (*r* − 1).

The PCC method is asymptotic, so no permutation is necessary to compute the *P*-value. For the WBC and SUP methods, we used an adaptive permutation procedure to compute the *P*-value (as coded by Hoffmann et al. [5]). We allowed the tests to have a maximum of 10,000 iterations, after which the *P*-value was said to be less than 0.0001 if no larger test statistics were observed.

Association at the gene-level was tested using the three methods on the 200 replicates of 209 case subjects and 488 control subjects and using the family-based PCC method on the 200 replicates of 194 nuclear families derived from the 8 extended families. In the case-control designs, we adjusted for age, sex, smoking, and ethnicity using the corresponding variable provided in the simulations (European, Denver Chinese, Han Chinese, Japanese, Luhya, Tuscan, and Yoruba). All analyses were undertaken using nonsynonymous variants only. Variants with a MAF below 5% were considered rare. In the results, we report the distributions of the *P*-values obtained for the top 30 genes ranked by median *P*-value. All analyses were performed without knowledge of the true simulated model.

## Results

### Genetic variants

Of the 24,487 variants detected through sequencing of the mini-exome, 21,355 (87%) had a MAF less than 5% and 18,131 (74%) had a MAF less than 1%. A total of 9,433 variants (39%) had a variant allele observed only once among the 697 genotypes (i.e., private alleles seen in only one individual). The proportion of nonsynonymous variants significantly increased when MAF decreased: 46.8% of variants with MAF greater than 5%, 50.5% of variants with MAF between 1% and 5%, and 60.2% of variants with MAF less than 1% (*P* = 4 × 10^−56^).

### Case-control design

**Figure 1:**
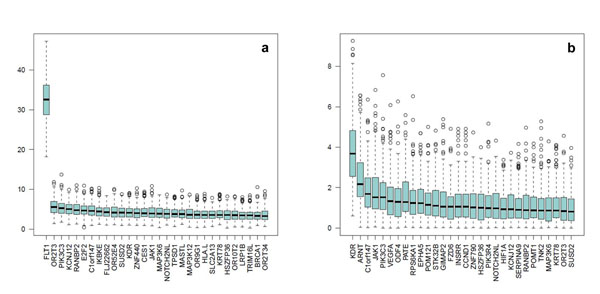
**Top 30 genes associated with Q1 using the principal components and collapsing method with a case-control design**. (a) Association with Q1 adjusting for age, sex, population, and smoking. (b) Association with Q1 adjusting for age, sex, population, smoking, and *FLT1*. Box plots represent the distribution of the 200 *P*-values of the 200 replicates for the 30 genes with the highest median *P*-values.

**Figure 2:**
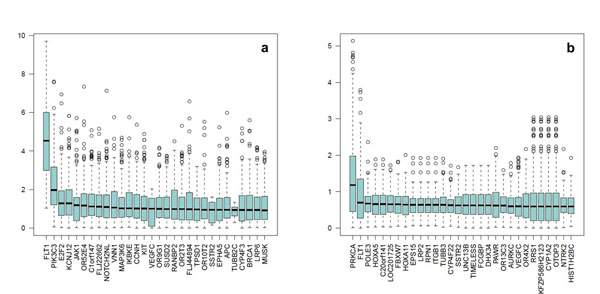
**Top 30 genes associated with disease phenotype using the principal components and collapsing method with a case-control design**. (a) Association with disease adjusting for age, sex, population, and smoking. (b) Association with disease adjusting for age, sex, population, smoking, and Q1. Box plots represent the distribution of the 200 *P*-values of the 200 replicates for the 30 genes with the highest median *P*-values.

**Figure 3:**
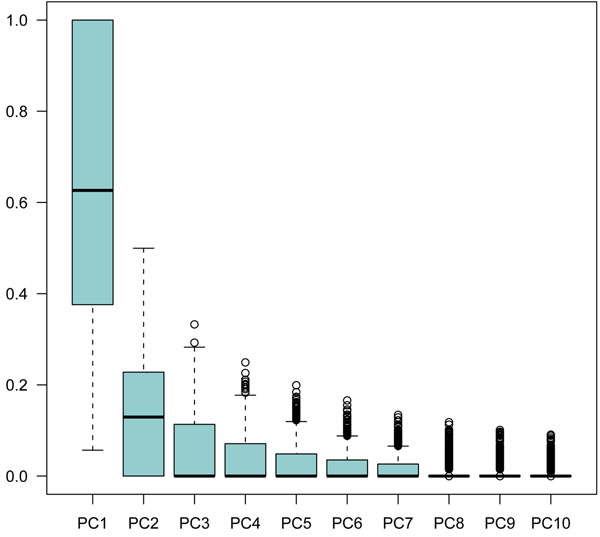
Proportion of variability within genes explained by each of the first 10 principal components

Using the PCC case-control method, we found an association of the principal component of gene *FLT1* with the quantitative trait Q1 (median *P*<10^−30^) (Figure 1a) and with the dichotomous disease phenotype (median *P*~10^−4^) (Figure 2a). However, when adjusting for Q1, the association with the dichotomous phenotype completely disappeared (Figure 2b). We also looked at the association with Q1 conditioning on the signal of *FLT1* and found that all the median *P*-values are reduced except for the gene *KDR*, which maintains a median *P*-value close to 10^−4^ (Figure 1b). The first principal component explained on average about 60% of the genetic variability (Figure 3).

**Figure 4:**
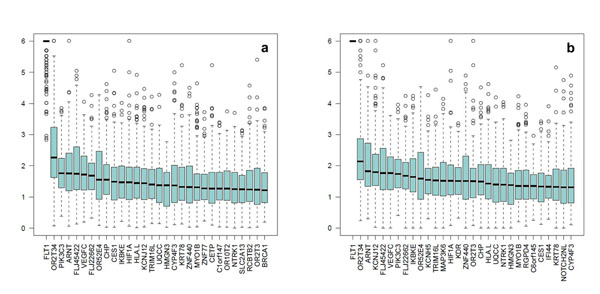
**Top 30 genes associated with Q1 using the weight-based and step-up methods with a case-control design**. (a) Weight-based method adjusting for age, sex, population, and smoking. (b) Step-up method adjusting for age, sex, population, and smoking. Box plots represent the distribution of the 200 *P*-values of the 200 replicates for the 30 genes with the highest median *P*-values.

The WBC and SUP methods lead to similar conclusions (Figure 4). *FLT1* is the gene with the highest median *P*-value (>10^−6^) and is the only gene that is statistically significant when a stringent Bonferroni correction for multiple testing is applied. In the top 30 list of genes discovered by the three methods using the case-control data sets, 13 genes are common to the three methods, 4 are common to the PCC and WBC methods, and 3 are common to the PCC and SUP methods. Moreover, the WBC and SUP methods have 10 genes in common that do not appear in the top 30 list of the PCC method. The PCC, WBC, and SUP methods ranked in their top 30 lists two, four, and five genes, respectively of the eight genes that had a true effect on Q1. Table 1 reports the average *P*-values over the 200 replicates obtained by the three methods for the nine genes with an effect on Q1.

**Table 1:** Average *P*-values obtained with the three methods for the nine genes containing variants with an effect on phenotype Q1 over 200 replicates

Gene	Number of variants		Principal components and collapsing method	Weight-based collapsing method	Step-up method
				
	Total	With effect			
*ARNT*	18	5	0.044	0.066	0.041
*ELAVL4*	10	2	0.177	0.303	0.388
*FLT1*	35	11	4 × 10^−21^	<10^−5^	<10^−5^
*FLT4*	10	2	0.132	0.182	0.186
*HIF1A*	8	4	0.008	0.091	0.074
*HIF3A*	21	3	0.51	0.488	0.320
*KDR*	16	11	0.003	0.587	0.059
*VEGFA*	6	1	0.289	0.2345	0.262
*VEGFC*	1	1	0.054	0.067	0.080

We compared the run times of the three approaches used on the case-control data sets. Testing the association of phenotype Q1 with all genes on chromosome 1 (where no markers were significant) took 4 s with the PCC method, 4 s with the WBC method, and 63 s with the SUP method, whereas on chromosome 13 (where *FLT1* had a *P*-value less than 0.0001), the PCC method took 1 s, the WBC method took 170 s, and the SUP method took 6,079 s.

### Family-based design

**Figure 5:**
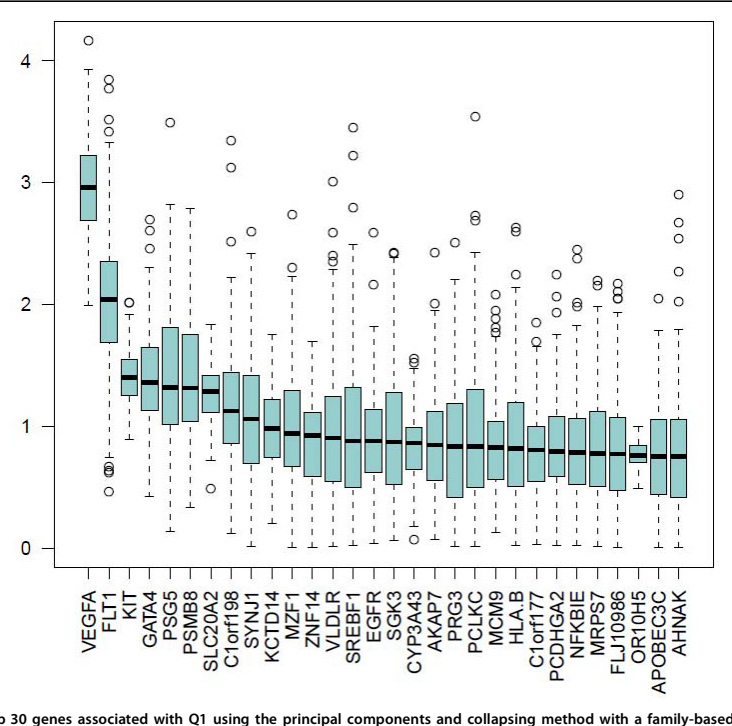
**Top 30 genes associated with Q1 using the principal components and collapsing method with a family-based design**. Box plots represent the distribution of the 200 *P*-values of the 200 replicates for the 30 genes with the highest median *P*-values.

Using the family-based PCC method, none of the top 30 genes had a median *P*-value less than 10^−3^ (Figure 5). *VEGFA* and *FLT1* ranked first and second with median *P*-values of 10^−2^ and 10^−3^, respectively.

## Discussion

The case-control and family-based PCC methods provide a computationally fast method of simultaneously testing rare and common variants and adjusting for covariates. Using a case-control design, the results obtained with the GAW17 simulations were similar to the results obtained using the WBC and SUP methods. The PCC methods are also much faster than the other two permutation-based approaches. Comparing our results to the true model used for simulating the data, we found that genes *FLT1* and *KDR* are indeed part of the gene list that controls the quantitative trait Q1. These genes have a common variant (MAF equal to 0.07 and 0.16, respectively) with a moderate or small effect (odds ratio of 1.92 and 1.15, respectively) and nine rare variants, each with an odds ratios ranging from 1.17 to 2.9. Some of the other genes controlling Q1 are represented in the top 30 lists obtained with the three methods, but none reaches significance when correction for multiple testing is implemented.

Although the top two genes (*VEGFA* and *FLT1*) obtained with the family-based association test using the PCC method are in the list of genes controlling Q1, their test statistics are not significant. This can be explained by the simulation procedures used for the GAW17 family data sets, which consisted of eight extended families. Our analysis split the eight pedigrees into 194 nuclear families and assumed that the nuclear families were independent.

Using only the first principal component seemed to be a reasonable choice where adjustment on multiple covariates was needed. However, the first principal component accounted on average for 60% of the variability within the genes. In practice, one might choose to include even more principal components to capture more genetic variability. The impact of this on power would be interesting to evaluate in future studies.

A strength of the PCC approach is its ability to evaluate large numbers of variants that are not amenable to fitting with a conventional logistic regression model. Another solution to this issue is to use shrinkage and variable selection methods, such as the least absolute shrinkage and selection operator (LASSO) [15]. Future work could compare the PCC method to these approaches.

In the PCA of each gene, the number of variables included was small (less than five in most cases) because rare variants were aggregated beforehand and only nonsynonymous variants were considered. However, for a larger number of variables, sparse PCA may provide a better alternative [16].

## Conclusions

In this paper, we propose a new method to analyze all variants within a predefined region based on the PCA of the collapsed rare variants term and all common variant terms. Applying this method to the simulated data sets of GAW17 provided results similar to two other methods with a greatly reduced computational time. However, evaluation of the statistical efficiency of these approaches is needed on a larger range of models and different family structures.

## Competing interests

The authors declare that there are no competing interests.

## Authors’ contributions

RK, TJH and JSW conceived the study. RK and TJH performed the statistical analysis. RK drafted the manuscript. TJH and JSW revised the manuscript. All authors read and approved the final manuscript.
